# Mechanism of Wheat Leaf Rust Control Using Chitosan Nanoparticles and Salicylic Acid

**DOI:** 10.3390/jof8030304

**Published:** 2022-03-16

**Authors:** Mohsen Mohamed Elsharkawy, Reda Ibrahim Omara, Yasser Sabry Mostafa, Saad Abdulrahman Alamri, Mohamed Hashem, Sulaiman A. Alrumman, Abdelmonim Ali Ahmad

**Affiliations:** 1 Agricultural Botany Department, Faculty of Agriculture, Kafrelsheikh University, Kafr Elsheikh 33516, Egypt; 2 Wheat Diseases Research Department, Plant Pathology Research Institute, Agricultural Research Center, Giza 12619, Egypt; redaomara43@gmail.com; 3 Department of Biology, College of Science, King Khalid University, Abha 62529, Saudi Arabia; ysolhasa1969@hotmail.com (Y.S.M.); amri555@yahoo.com (S.A.A.); drmhashem69@yahoo.com (M.H.); salrumman@kku.edu.sa (S.A.A.); 4 Department of Botany and Microbiology, Faculty of Science, Assiut University, Assiut 71515, Egypt; 5 Department of Plant Pathology, Faculty of Agriculture, Minia University, El Minia 61519, Egypt; abdelmonim.ali@mu.edu.eg

**Keywords:** *Puccinia triticina*, wheat, salicylic acid, chitosan nanoparticles, enzymes, ROS, anatomical characters

## Abstract

Wheat leaf rust is one of the world’s most widespread rusts. The progress of the disease was monitored using two treatments: chitosan nanoparticles and salicylic acid (SA), as well as three application methods; spraying before or after the inoculation by 24 h, and spraying both before and after the inoculation by 24 h. Urediniospore germination was significantly different between the two treatments. Wheat plants tested for latent and incubation periods, pustule size and receptivity and infection type showed significantly reduced leaf rust when compared to untreated plants. *Puccinia* *triticina* urediniospores showed abnormalities, collapse, lysis, and shrinkage as a result of chitosan nanoparticles treatment. The enzymes, peroxidase and catalase, were increased in the activities. In both treatments, superoxide (O_2_^−^) and hydrogen peroxide (H_2_O_2_), were apparent as purple and brown discolorations. Chitosan nanoparticles and SA treatments resulted in much more discoloration and quantitative measurements than untreated plants. In anatomical examinations, chitosan nanoparticles enhanced thickness of blade (µ), thickness of mesophyll tissue, thickness of the lower and upper epidermis and bundle length and width in the midrib compared to the control. In the control treatment’s top epidermis, several sori and a large number of urediniospores were found. Most anatomical characters of flag leaves in control plants were reduced by biotic stress with *P. triticina*. Transcription levels of *PR1-PR5* and *PR10* genes were activated in chitosan nanoparticles treated plants at 0, 1 and 2 days after inoculation. In light of the data, we suggest that the prospective use of chitosan nanoparticles might be an eco-friendly strategy to improve growth and control of leaf rust disease.

## 1. Introduction

Wheat is one of Egypt’s and the world’s most vital nutritional winter crops. Stripe, stem and leaf rust diseases induced by *Puccinia striiformis* f.sp. *tritici*, *P. graminis* f.sp. *tritici*, and *P. triticina* f.sp. *tritici*, respectively, may attack wheat throughout the season [1]. Leaf rust is Egypt’s most frequent disease of wheat. It appears annually on wheat varieties and causes annual losses in grain yield. Several epidemics of leaf rust on wheat crop have been reported in the past, and this disease is a major hazard to future wheat production [2]. Moreover, it results in a significant reduction in grain production, which may be as high as 23% on susceptible wheat cultivars under ideal climatic conditions [2,3]. Since the fungus is an obligate parasite, it can continuously produce infectious urediniospores. Urediniospores are spread by the wind across great distances, infecting new wheat crops in the spring. For infection and disease development, temperatures between 10 and 18 °C with six h of dew are ideal. A new generation of pustules and spores may emerge every two weeks under these conditions [2]. The treatments with some chemical compounds and pre–inoculation with beneficial microbes may be used to systematically generate resistance in certain susceptible plants [4]. Tilt and Crown 25% fungicides were utilized in the management of yellow rust disease and showed excellent results in reducing disease severity [4]. However, the way the farmers deal with the fungicide may have a negative impact on their lives, and there can be residual effects of fungicides on the environment.

Wheat leaf rust disease has yet to be cured; therefore, the first line of defense in the resistance is the cultivation of resistant varieties. In recent years, the cultivation of more than one resistant variety was utilized [1]. The emergence of new disease races and the breaking of resistance in the varieties increase disease losses [5]. Therefore, the second line of disease control is to rely on chemical control. However, chemical control causes pollution to the environment, humans and animals. Furthermore, using the same fungicides regularly may raise the possibility of establishing aggressive fungicide-resistant strains [6]. Additionally, increased fungicide use has negative impacts on human health, food safety, and environmental hazards, as well as the potential for toxicity to non-target beneficial bacteria. As a result, fungicide-based management methods are not long-term effective. This encourages us to search for some safe alternative methods to combat this disease. Compounds such as, benzothiadiazole (BTH), other chemical inducers, *Artemisia cina* extract, salicylic acid and chitosan were used to control several diseases [7,8,9,10]. However, the effect of chitosan nanoparticles on the severity of wheat rust diseases and the involved mechanisms in disease resistance remain unclear. Sustainable techniques are needed to develop innovative alternative control approaches that combine safe and environmentally acceptable methods such as chitosan nanoparticles and salicylic acid to decrease the use of fungicides completely or partly. 

Bacteria, fungi, and viruses are often associated with the buildup of reactive oxygen species (ROS), which causes oxidative stress in plants. Up-regulation of antioxidant defense mechanisms in plants appears to be a general reaction to oxidative stress under natural circumstances [6]. However, plant cells produce different antioxidant enzymes to reduce the harmful effects of oxidation. Superoxide dismutase, which converts superoxide (O_2_^−^) into hydrogen peroxide (H_2_O_2_), and catalase, which converts H_2_O_2_ into water and oxygen gas, are two key players [11]. O_2_^−^ and H_2_O_2_ have the potential to degrade DNA, proteins, and lipids, making them toxic to the pathogen. ROS metabolism during pathogen attack includes several antioxidant enzymes such as ascorbate peroxidase (APX), peroxidase (POX), superoxide dismutase (SOD), catalase (CAT), polyphenol oxidase (PPO), and glutathione reductase (GR). Plants may be protected from oxidative stress by developing an antioxidant defense mechanism that detoxifies ROS [9,11]. 

Nanotechnology is regarded as a vital method having economic, social, and environmental implications [12,13,14]. The area of nanotechnology is one of the most active fields of recent research [15]. Nanoparticles are defined by certain features such as size, shape and distribution with new or improved characteristics [16]. Nanoparticles and nanomaterials are swiftly used for novel applications. Nanotechnology is now being utilized in today’s antimicrobial industry [17]. Chitosan is a naturally present cationic biopolymer consisting of N-acetyl-D-glucosamine and D-glucosamine units linked together by β-1,4-glycosidic bonds [18,19]. A previous study has evaluated the antibacterial properties of chitosan, and more recently, several kinds of chitosan derivates have been made to boost its natural antimicrobial properties [20,21]. Moreover, chitosan treatment affects a number of genes in plants, including the genes involved in defense pathway activation, resulting in the accumulation of defense proteins [22].

The research aims to test the potential of chitosan nanoparticles and salicylic acid against wheat leaf rust disease. A variety of immune-related responses to chitosan nanoparticles and salicylic acid treatments (before inoculation, after inoculation and before and after inoculation) was investigated. Therefore, the effects of chitosan nanoparticles and salicylic acid on stimulating systemic resistance (activation of CAT, POX and ROS) and transcription levels of defense-related genes, as well as the direct effect on urediniospores were evaluated to determine how chitosan nanoparticles and salicylic acid affect rust disease. 

## 2. Materials and Methods

This research was performed in the leaf rust greenhouse, ARC, Giza, Egypt (20–22 °C, 14/10 light/dark cycle, 50–55% relative humidity). Chitosan nanoparticles (purity 99%, Nanoshel, Congleton, UK) were mixed with acetic acid (1%) and held overnight under magnetic stirring for the full dissolving of its particles before diluting with distilled water to get the appropriate volume. The concentration of nano chitosan and salicylic acid is 150 µg/ml. The same concentration of acetic acid (1%) was mixed with water for the control treatment. Under greenhouse conditions, the effects of chitosan nanoparticles and salicylic acid treatments on the incidence of leaf rust disease were studied.

### 2.1. Cultivation of Wheat Plants

Morocco, a susceptible variety, was cultivated in plastic pots (10 cm in diameter, filled with clay soil) using 10 grains per pot in the greenhouse. The inoculation and incubation procedures were performed after 7 days of planting [23]. To induce spore germination and development of infection, seedlings were rubbed carefully between wet fingers with water, and infected samples were scraped using sterile spatulas and applied to these seedlings and carefully sprayed again with water. Finally, the infected seedlings were incubated for 24 h in moist chambers at 18–20 °C and 100% RH before being transferred to benches in a greenhouse for 14 days at a temperature 20 ± 2 °C with 50–55% relative humidity and 7500 Lux light intensity (14 h light and 10 h dark). Twelve days after pustules appeared, rust data were collected. After pustules rupture, rust data were recorded after 12 days. Rust symptoms were graded as infection type, with resistance (=0, 0; 1 and 2) and susceptible (=3 and 4) indicating low infection type and high infection type, respectively (Supplementary Table S1) [24]. The application methods were A= spray 24 h before inoculation, B = spray 24 h after inoculation C = spray 24 h before and after inoculation. Chitosan nanoparticles and salicylic acid treatments were sprayed at a rate of 10 mL per plant.

### 2.2. Morphogenesis of the Disease on the Susceptible Variety 

#### 2.2.1. Effect of Chitosan Nanoparticles and Salicylic Acid Treatments on *Puccinia triticina* Spores

Chitosan nanoparticles and salicylic acid treatments were sprayed directly on the susceptible variety, then (after 6 h) leaf samples (6 cm long) were chopped and observed under a light microscope to observe the morphology of the spores. 

On glass slides, urediniospores were placed according to the general method [25]. Slides were washed with ethyl alcohol and air-dried before being covered with a thin smear of 2% water agar that had been supplemented with chitosan nanoparticles, salicylic acid and non-treated control. In sterilized Petri dishes containing many layers of water-saturated filter papers, slides were put on V-shaped glass rods. Slides holding spores were incubated at 25 °C for 12 h under continuous illumination before being examined microscopically at ×100 magnification to evaluate spore germination [26]. A germ tube longer than the spore’s width was considered valid for spores’ germination [27]. For 100 spores on a slide, germination percentages were determined. For each treatment, three slides were analyzed. Water agar slides without treatments were used as a control.

#### 2.2.2. Latent and Incubation Periods and Number of Pustules 

Incubation period was determined by counting the number of visible pustules on marked leaves per day until no more pustules developed [28]. The latent period was determined by the time between inoculation and 50% of pustules that were evident or emerged. On the top surface of the leaves, the number of pustules per unit leaf area (2.0 × 0.5 cm^2^) was counted [29]. 

#### 2.2.3. Measurement of Pustule Size 

Due to the evident variable forms of *P. triticina* pustules, length and width measurements were used to compare pustule sizes. The dimensions of 20 pustules on the first leaf of susceptible plants were assessed in length and width. 

### 2.3. Biochemical Assays of Antioxidant Enzymes 

Fresh wheat leaf material (0.5 g) was homogenized in 50 mM Tris buffer (3 mL, pH 7.8, with 1 mM EDTA-Na2) and 7.5% polyvinylpyrrolidone for enzyme analyses. The homogenates were centrifuged at 12,000 rpm for 20 min [8]. The UV-160A spectrophotometer (Shimadzu, Japan) was used for all measurements, which were performed at 25 °C. Catalase activity was measured [30]. Sodium phosphate buffer (2 mL, 0.1 M, pH 6.5), 100 µL of H_2_O_2_ (0.02M) and enzyme extract (50 µL) were added to the reaction mixture. The activity was estimated using the extinction coefficient (0.04 mM^−1^ cm^−1^ at 240 nm). For 3 min, changes at 240 nm absorbance were measured every 30 sec. Molecular hydrogen peroxide g FW^−1^ min^−1^ was used to measure enzyme activity.

Peroxidase was assessed in the crude enzyme extract [31]. Sodium phosphate buffer (2 mL, pH 6.0) with 0.25% (V/V) guaiacol (2-methoxyphenol, 100 μL) and 100 mM H_2_O_2_ (100 μL) were used in the process. A crude enzyme extract (100 μL) was added to start the procedure. For 3 min, the changes in the absorbance (470 nm) were determined at 30 s intervals. The tetra-guaiacol extinction coefficient (26.6 mM^−1^ cm^−1^ at 470 nm) was employed to measure the activity. The activity of enzymes was measured in µmol tetraguaiacol g FW^−1^ min^−1^.

### 2.4. Histochemical Analysis of Reactive Oxygen Species (ROS) 

Nitro blue tetrazolium (NBT) and 3,3-diaminobenzidine (DAB) were used to detect superoxide (O_2_^−^) and hydrogen peroxide (H_2_O_2_), respectively. Infiltration of the leaves was done with potassium salicylate buffer (10 mM, pH 7.8, containing 0.1% NBT or DAB, Sigma–Aldrich, Steinheim, Germany). To remove the trichloroacetic acid, the samples were purified in ethanol:chloroform (4:1, *v*/*v*) for a day using trichloroacetic acid in NBT-and DAB-treated samples (0.15 *w*/*v*%) [32]. Before being evaluated, cleared samples were rinsed with water and transferred into 50% glycerol. Using Chemilmager 4000 digital system, discoloration of leaves was measured.

### 2.5. Anatomical Studies

At the age of 15 days, flag leaves measuring 0.5 cm in length were collected. The materials were cleaned in 50% ethyl alcohol and dehydrated in a standard butyl alcohol series after treatment with the killing and fixation solution (FAA). The specimens were then coated in paraffin wax (56–58 °C). The rotary microtome type 820 was used to cut transverse sections that were 12 microns thick. Albumin was used to fix the pieces, which were then dyed with safranin and mounted in canada balsam [33]. The sections were inspected microscopically and photographed.

### 2.6. Defense-Related Genes Transcriptional Levels

Wheat samples (the first leaf) were used to extract RNA. For all treatments, 100 mg of wheat leaves were collected at 0, 1, and 2 dpi (days post inoculation) for total RNA extraction. RNA was purified using the kit Thermo Scientific, Fermentas, #K0731 [34]. After verifying the concentration, integrity and purity of RNA were assessed using agarose gel electrophoresis and Nano SPECTROstar. The reverse transcription process was done using the reverse transcription kits (Thermo Scientific, Fermentas, #EP0451). The generated cDNA was employed for qRT-PCR amplification using specific primers to identify the expression patterns of the six wheat genes (*PR1-PR5* and *PR10*) (Table 1) [35]. All genes transcripts were amplified using a real-time cycler [35]. To normalize gene expressions, the *β-tubulin* reference gene (Table 1) was used. Three technical and biological replicates were used for each treatment. The relative expression levels were calculated using Livak and Schmittgen’s method [36].

### 2.7. Statistical Analysis

Three repetitions of a randomized complete block design (RCBD) were utilized. Analysis of variance (ANOVA) was used to statistically examine the data using the SPSS software V22.0 22 (SPSS Inc., Chicago, IL, USA).

## 3. Results

### 3.1. Effect of Chitosan Nanoparticles and Salicylic Acid on Urediniospores Germination

**Figure 1:**
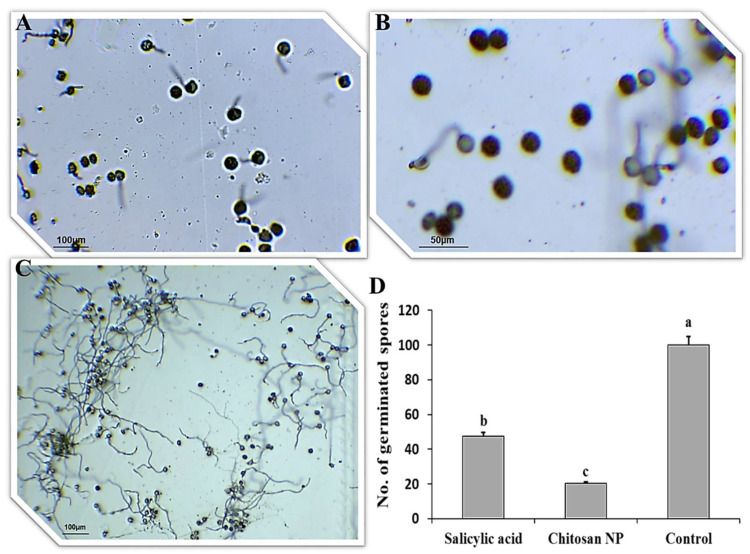
*Puccinia triticina* spore germination on water agar media with two treatments; salicylic acid (**A**), chitosan nanoparticles (**B**) and control non-treated (**C**) and the number of germinated spores (**D**). The letters (a, b and c) denote significant difference.

The effect of salicylic acid and chitosan nanoparticles on *P. triticina* urediniospores germination on water-agar medium was studied (Figure 1). All treatments gave significant differences in urediniospores germination. The best treatment was chitosan nanoparticles in the inhibition of germination (Figure 1A,D), followed by salicylic acid treatment (Figure 1B,D) compared to the control (Figure 1C,D).

### 3.2. The Effect of Chitosan Nanoparticles and Salicylic Acid on the Development of Wheat Leaf Rust 

**Figure 2:**
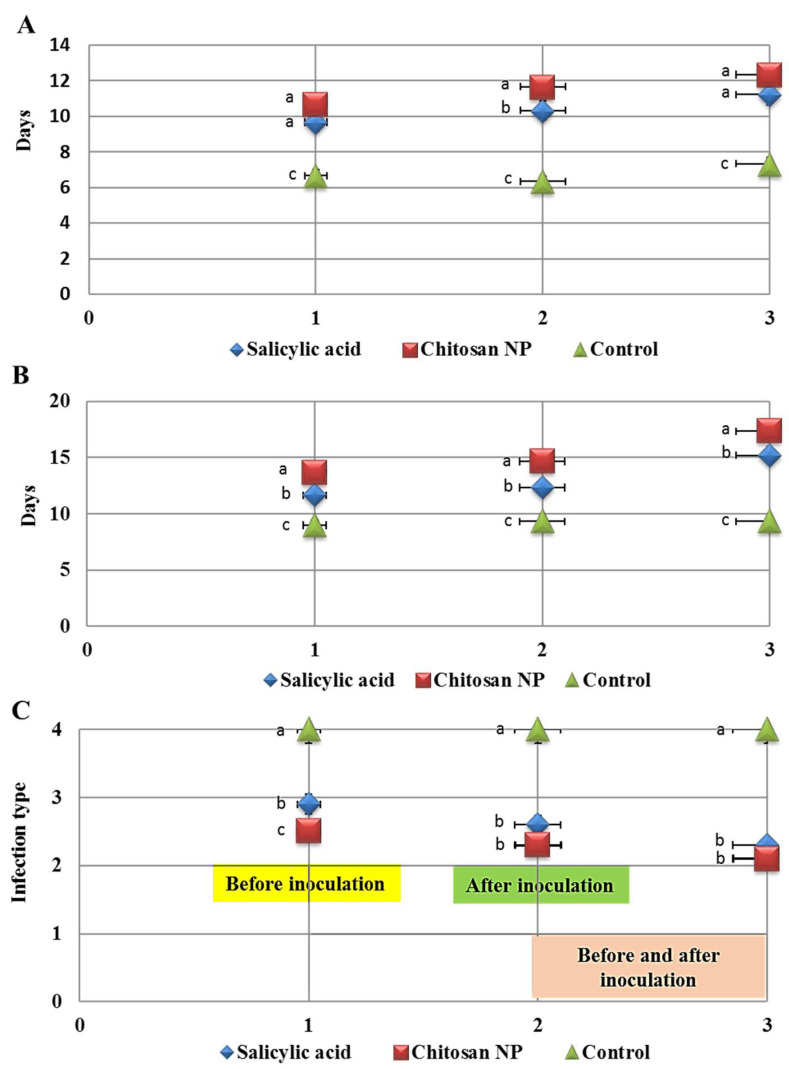
Effect of salicylic acid and chitosan nanoparticles application methods (1 = spray before inoculation by 24 h, 2 = spray after inoculation by 24 h and 3 = spray before and after inoculation by 24 h) on incubation period (**A**), latent periods (**B**) and infection type (**C**) of wheat leaf rust disease. The letters (a, b and c) denote significant difference.

**Figure 3:**
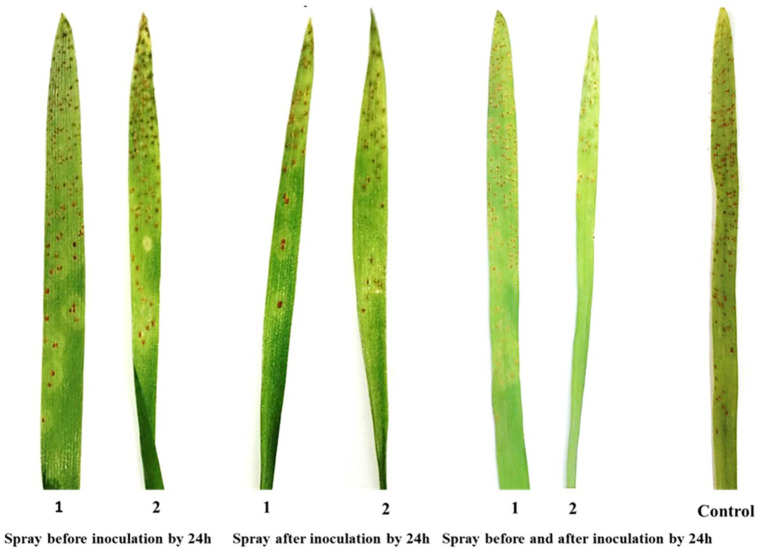
Effect of salicylic acid (1) and chitosan nanoparticles (2) applications (spray before inoculation by 24 h, spray after inoculation by 24 h and spray before and after inoculation by 24 h) on infection type of wheat leaf rust disease.

Data illustrated in (Figure 2) show the effect of salicylic acid and chitosan nanoparticles and three application methods; spray before inoculation by 24 h, spray after inoculation by 24 h and spray before and after inoculation by 24 h on incubation period, latent period and infection type to assess disease development on treated and untreated wheat plants. The two treatments were significantly effective in controlling leaf rust compared with the check control (treated with water). Chitosan nanoparticles treatment revealed a substantial rise in latent and incubation periods compared to the control treatment, which displayed the highest latent and incubation periods with three application methods (Figure 2A,B). Salicylic acid significantly increased incubation and latent periods compared to the control treatment but with less order. On the other hand, chitosan nanoparticles treatment decreased infection type (IT) with three applications compared to control (Figure 2C and Figure 3). It was also noted that the best application method was spray before and after inoculation by 24 h on increased incubation and latent periods and decreased infection type, followed by the application method of spray after inoculation by 24 h.

### 3.3. Effect of Chitosan Nanoparticles and Salicylic acid on Pustules Size and Receptivity

**Figure 4:**
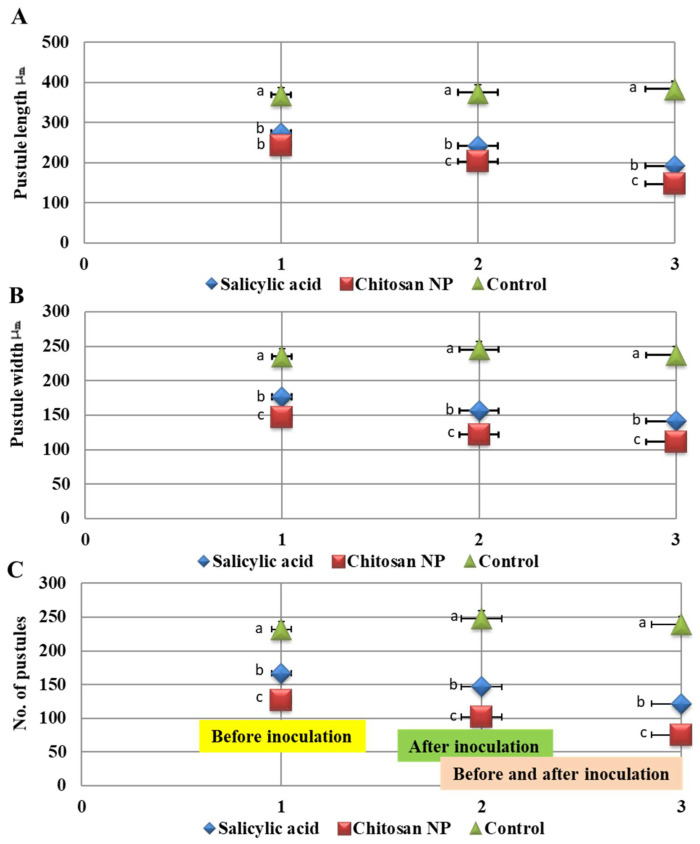
Effect of salicylic acid and chitosan nanoparticles applications (1 = spray before inoculation by 24 h, 2 = spray after inoculation by 24 h and 3 = spray before and after inoculation by 24 h) on pustules length (**A**), pustules width (**B**) and receptivity (**C**). The letters (a, b and c) denote significant difference.

It was clear from the study that chitosan nanoparticles and salicylic acid achieved significant results in influencing the pustule size (pustules length and width) and the number of pustules compared to the control with the three application methods (Figure 4). Chitosan nanoparticles treatment was the best treatment in reducing pustules length and width compared to the control, followed by salicylic acid treatment (Figure 4A,B). Similarly, chitosan nanoparticles treatment was the best treatment in decreasing the number of pustules (1 cm^2^) compared to the control (Figure 4C). However, spray before and after inoculation by 24 h was the best application method. 

### 3.4. Effect of Chitosan Nanoparticles and Salicylic Acid on Disease Symptoms

**Figure 5:**
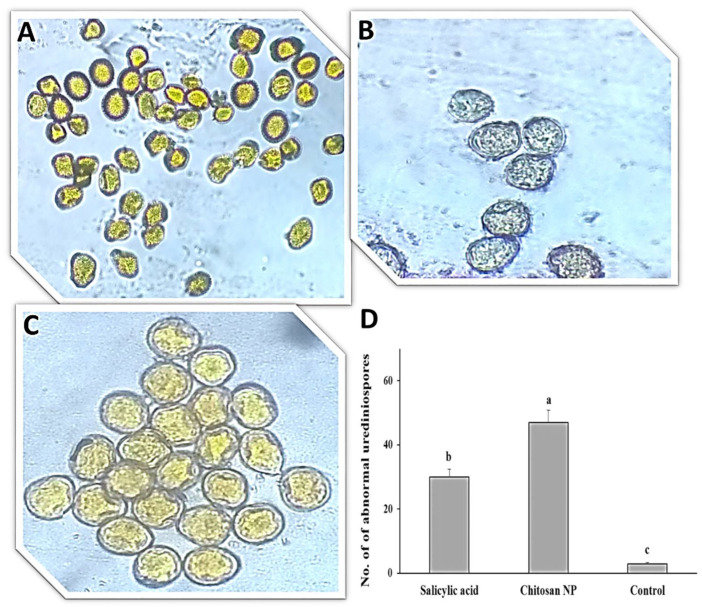
Light microscopy of *P. triticina* spores on wheat leaves treated with salicylic acid (**A**), chitosan nanoparticles (**B**) and control (untreated) (**C**) and the number of collapses, lysis, abnormalities and shrinking of urediniospores (**D**). The letters (a, b and c) denote significant difference.

The direct effect of the tested treatments on symptoms was studied (Figure 5). The effect was evident through abnormalities, collapse, lysis and shrinking of urediniospores in *P. triticina*. Chitosan nanoparticles treatment resulted in lysis, abnormalities and shrinking of urediniospores (Figure 5A), while salicylic acid treatment resulted in abnormalities, collapse and shrinking of urediniospores (Figure 5B) compared to the control (Figure 5C). Moreover, the number of abnormal urediniospores was high with chitosan nanoparticles followed by salicylic acid compared to the control (Figure 5D).

### 3.5. Effect of Chitosan Nanoparticles and Salicylic Acid Treatments on Enzyme Activities

**Figure 6:**
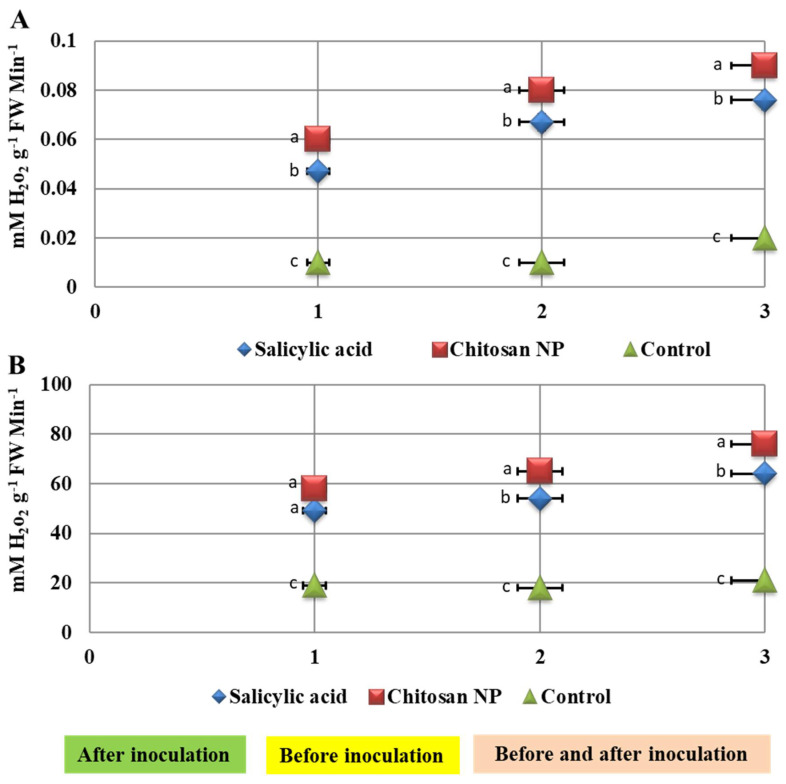
Effect of salicylic acid and chitosan nanoparticles application methods (1 = spray before inoculation by 24 h, 2 = spray after inoculation by 24 h and 3 = spray before and after inoculation by 24 h) on peroxidase (**A**) and catalase (**B**) activities in wheat leaves infected with *Puccinia triticina*. The letters (a, b and c) denote significant difference.

The activity of the enzymes represented by peroxidase and catalase was demonstrated with different application methods (Figure 6). The highest values were achieved in before and after inoculation by 24 h followed by after inoculation by 24 h in increasing the activity of enzymes. The study also showed that the maximum increase in enzyme activities was achieved using chitosan nanoparticles, followed by salicylic acid, and all this compared to control (Figure 6).

### 3.6. Histochemical Analysis of Reactive Oxygen Species (ROS)

**Figure 7:**
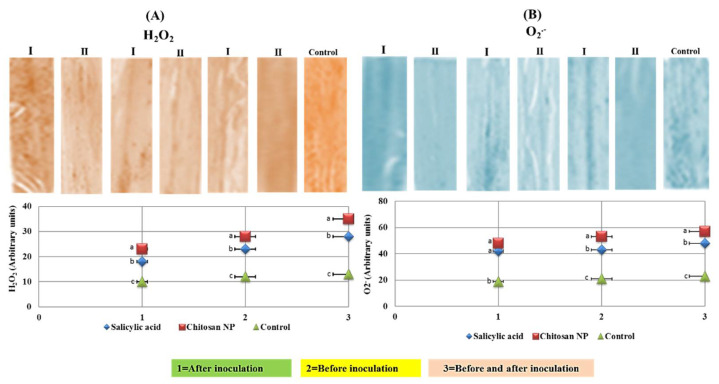
Effect of salicylic acid (I) and chitosan nanoparticles (II) on hydrogen peroxide (**A**) and superoxide anion (**B**) in infected plants with *Puccinia triticina* with three application methods (1 = spray before inoculation by 24 h, 2 = spray after inoculation by 24 h and 3 = spray before and after inoculation by 24 h). The letters (a, b and c) denote significant difference.

Hydrogen peroxide (H_2_O_2_) and superoxide (O_2_^−^) were visualized as purple and brown discoloration in the salicylic acid and chitosan nanoparticles treatments and they were also quantified (Figure 7). The discoloration was significantly increased in chitosan nanoparticles treatment compared to the control treatment (Figure 7A). For quantitative measurements, the chitosan nanoparticles treatment was the highest in hydrogen peroxide (H_2_O_2_), and superoxide (O_2_^−^) compared to the control treatment (Figure 7B). Moreover, the highest values of all treatments were achieved in the application method of before and after inoculation by 24 h.

### 3.7. Effect of Chitosan Nanoparticles and Salicylic Acid on Anatomical Traits

**Figure 8:**
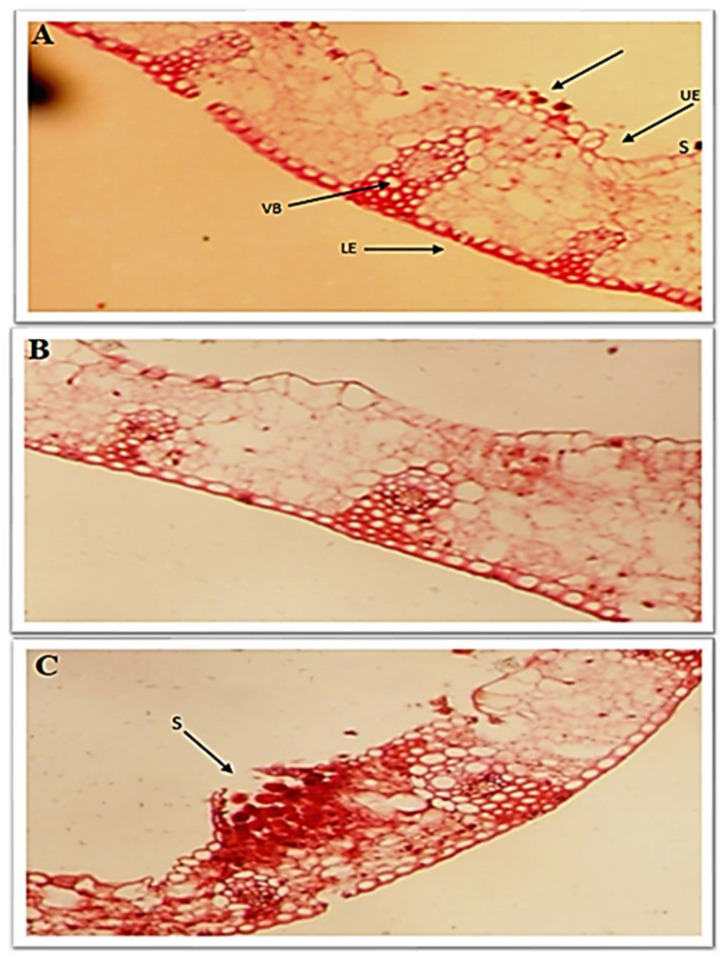
Transverse sections of salicylic acid (**A**) and chitosan nanoparticles (**B**) compared to the control (**C**). (Magnification × 100). Details:—UE: upper epidermis, VB: vascular bundle, MT: mesophyll tissue, S: urediniospores.

The best application method was chosen through the previous results to evaluate the effect of the treatments on anatomical traits of infected wheat leaves. Data illustrated that flag leaf anatomical traits were reduced in control plants exposed to *P. triticina* stress (Figure 8). Salicylic acid and chitosan nanoparticles treatments increased the thickness of blade (µ), the thickness of the lower and upper epidermis, the thickness of mesophyll tissue and bundle length and width in the midrib compared to the control. Additionally, urediniospores and sori are abundant in the upper epidermis of the control leaves (Figure 8). The impact of chitosan nanoparticles treatment was higher than salicylic acid treatment in decreasing the number of urediniospores and increasing the thickness of blade (µ), the thickness of the lower and upper epidermis, the thickness of mesophyll tissue and bundle length and width in the midrib (Figure 8). 

## 4. Defense-Related Genes Transcriptional Levels

**Figure 9:**
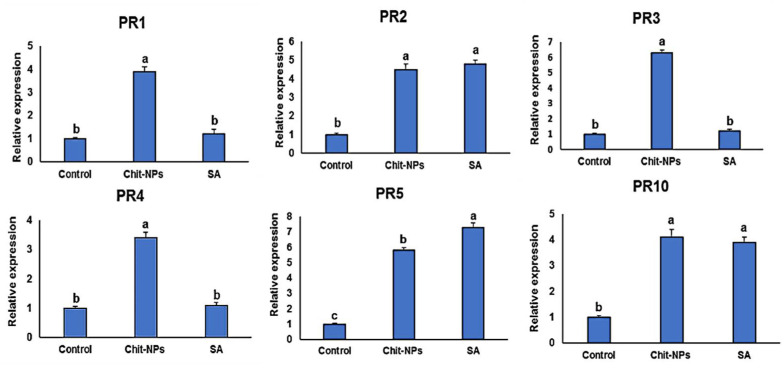
Effect of chitosan nanoparticles (Chit-NPs) and salicylic acid (SA) on the relative transcription levels of *PR1-PR5* and *PR10* genes in infected wheat plants at 1 day after treatment (0-day post inoculation). The letters (a, b and c) denote significant difference.

**Figure 10:**
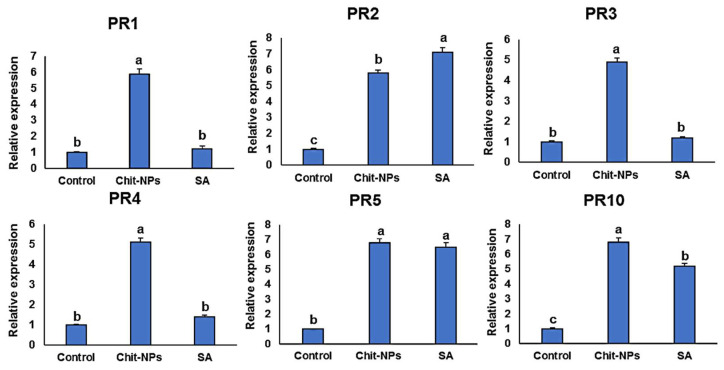
Effect of chitosan nanoparticles (Chit-NPs) and salicylic acid (SA) on the relative transcription levels of *PR1-PR5* and *PR10* genes in infected wheat plants at 1 day post inoculation. The letters (a, b and c) denote significant difference.

**Figure 11:**
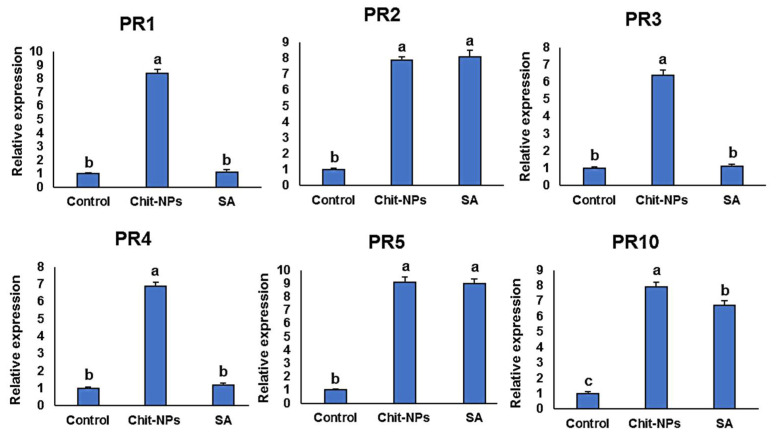
Effect of chitosan nanoparticles (Chit-NPs) and salicylic acid (SA) on the relative transcription levels of *PR1-PR5* and *PR10* genes in infected wheat plants at 2 days post inoculation. The letters (a, b and c) denote significant difference.

Pathogenesis-related genes (*PR1-PR5* and *PR10*) transcription levels were examined at time intervals of 0, 1 and 2 days after inoculation. One day after treatments (0 day after inoculation), induction of the genes *PR1*, *PR3* and *PR4* was significantly greater in the chitosan nanoparticles treated plants compared to salicylic acid-treated plants, and no significant differences was found in the case of *PR2* and *PR10* gene expressions (Figure 9). One day after inoculation, the genes *PR1*, *PR3, PR4* and *PR10* were significantly stimulated to higher levels in chitosan nanoparticles treated plants, and no significant difference was found between both treatments in the case of *PR5* (Figure 10). This is principally striking for *PR3* and *PR4*, which had transcription levels around 5 times higher in the chitosan nanoparticles treatment than in salicylic acid treatment. *PR1, PR3, PR4* and *PR10* had the greatest expression levels in chitosan nanoparticles at 2 dpi when compared to salicylic acid and a mock-inoculated control (Figure 11). Both chitosan nanoparticles and salicylic acid dramatically increased transcriptions of *PR2* and *PR5*, with relative expression levels almost 8-fold higher than the mock-inoculated control.

## 5. Discussion

Leaf rust is a common disease in wheat in all growing areas of the world. Field observations showed that it appears annually at varying magnitudes in the different areas [3]. The severity of infection varies according to the sensitivity of each variety to this disease. Therefore, in the case of planting a highly susceptible variety, chemical resistance must be used to reduce the resulting losses [37]. This can also be achieved by developing rust resistant genotypes or by successful varietal manipulation of the available genotypes throughout the country to avoid heavy infection to the susceptible genotypes [1]. The emergence of new races capable of breaking the resistance in the new varieties, such as the emergence of the TTTST race on Shandweel-1, Sakha-94 and Sakha-95 varieties was reported [5]. Therefore, the second line of control methods should be used, which is chemical fungicides. The use of these chemical fungicides results in pollution to the environment and is dangerous to humans, especially people who are not familiar with the precautionary measures to deal with pesticides [38]. Therefore, the study turned to the use of some safe materials to combat this disease, such as salicylic acid and chitosan nanoparticles [10]. The effect of these chemicals on urediniospores germination and disease development was evaluated. All treatments gave significant differences in urediniospores germination. The best treatment in increasing the incubation and latent periods was chitosan nanoparticles. Plant pathogens have been reported to exhibit chitinolytic and chitosanolytic activities [39]. Variations in chitosan’s impact on hyphal development were discovered in a previous study across nine plant pathogens, although *V. dahliae* was the most tolerant among them [40]. 

Chitosan nanoparticles treatment increased latent and incubation periods as well as decreased infection type, pustule size and the number of pustules compared to the control treatment. Salicylic acid was also effective in increasing latent and incubation periods and decreasing infection type, pustule size and number of pustules compared to the control treatment, but it had lower effectiveness compared to chitosan nanoparticles. The high effectiveness of chitosan and salicylic acid in inducing resistance against *Botrytis cinerea* under the plastic house was elucidated in a previous study [10]. The active role of chemical inducers such as BTH and salicylic acid in reducing the impact of sugar beet rust was reported [41]. Natural polymer chitosan is widely believed to be the most prevalent natural polymer with dual functions. It inhibits pathogen growth, viability, sporulation, germination and cell alterations, as well as stimulating and/or suppressing diverse defensive responses in host plants [42]. Previous studies confirmed that the application of chitosan led to a reduction in the pathological severity of root diseases [43,44]. Chitosan’s antifungal effect is associated with its capacity to interfere with the plasma membrane of fungal cells and fungal DNA and/or RNA [45,46]. The treatments also clearly increased the anatomical characters of flag leaves in wheat plants. Clear increases in the thickness of blade (µ), the thickness of the lower and upper epidermis, the thickness of mesophyll tissue and bundle length and width in the midrib compared to untreated plants (control) were observed. Lower concentrations of chitosan nanoparticles were more effective than higher concentrations. Chitosan nanoparticles improved seed germination, water uptake and transport, activation of water channels proteins, and increased the absorption of nutrients in the plant. All of these alterations disappeared when the concentration of chitosan nanoparticles was increased [47].

It was necessary to explain how chitosan nanoparticles and salicylic acid work in the development of the disease, by studying the activity of some enzymes such as peroxidase and catalase as well as by studying reactive oxygen species such as hydrogen peroxide (H_2_O_2_) and superoxide (O_2_^−^). The maximum increase in enzyme activities was achieved by chitosan nanoparticles, specially the application method of before and after inoculation by 24 h compared to the control. Salicylic acid treatment resulted in increases in chitinase and peroxidase activity on both the local and systemic levels [48]. Superoxide and hydrogen peroxide discolorations were significantly increased using chitosan nanoparticles and salicylic acid treatments compared to the control treatment. For quantitative measurements, chitosan nanoparticles treatment was the highest in superoxide and hydrogen peroxide compared to the control. Antioxidants, both non-enzymatic and enzymatic, effectively remove ROS in non-stressful situations however, under stress, the synthesis of ROS and antioxidant enzymes may be affected. ROS have recently been shown to be beneficial for biosystems as signaling molecules and immune defense stimulants [49]. ROS, on the other hand, have the potential to damage organs and tissues. Studies focusing on diseases linked to ROS are controversial. There are increasing numbers of studies showing that chitosan and its derivatives have several mechanisms of action. It suppresses pathogen development and alter the defensive response of host plants [50]. Salicylic acid plays an important role in signal transduction of resistance in various plant pathogen interactions. Salicylic acid activated various defense reactions in plants against pathogen [51]. Salicylic acid induces rapid transient-generation of reactive O_2_ through oxidative burst in incompatible interaction [10]. Significant increases in the activity of peroxidase and polyphenol oxidase were found after spraying wheat and sugar beet plants with salicylic acid [41,52].

Pathogenesis related proteins, β-1, 4-glucanase, peroxidase, and chitinase were activated in resistant plants [53]. Lignification in wheat seems to be of special value in induced resistance. Lignin biosynthesis in wheat is related to defense enzymes [54]. The increased lignification’s rate was reported through accomplished hypersensitive reaction due to foliar application of BABA [55]. The microbial defense-enhancing activities of chitinase and beta 1,3-glucanase were useful in developing resistance to fungi [56]. The transcript levels of *PR1-PR5* and *PR10* genes were assessed using RT-qPCR relative to the reference gene *β-tubulin.* Defense genes were activated after chitosan nanoparticles and salicylic acid treatments compared to mock-inoculated controls at all three time periods analyzed. After being treated with chitosan nanoparticles for 9 h, downy mildew-infected pearl millet plants showed increased levels of defense enzymes [57]. Many genes in plants are regulated by chitosan treatment, including the stimulation of phytoprotective pathways [22]. Chitosan has been shown to increase the defense enzyme activities in *Pinus koraiensis* seedlings to their maximum levels at 2 days post-inoculation (dpi) [58]. Peroxidase, phenylalanine ammonium lyase and pathogen-related protein-1 transcriptional levels were all shown to be associated with systemic resistance induction by chitosan nanoparticles. 

Through this study, it was found that salicylic acid treatment was less effective in reducing the development of the wheat leaf rust disease than chitosan nanoparticles, and this could be due to that salicylic acid required more time and high dosage before induction of resistance. The increased activities of peroxidase and catalase were associated with the induction of resistance. *PR1-PR5* and *PR10* gene transcriptions were considerably greater in plants treated with chitosan nanoparticles than in controls. Multiple mechanisms were established to mediate the resistance elicited by chitosan nanoparticles, resulting in a full decrease in the disease. The nano product of chitosan increased its effectiveness in the process of combating this disease. To manage plant pathogens in agricultural crops, nanotechnology seems to have great promise. For novel formulations of plant disease-control fungicides, concentration, molecular weight, particle size, and dose are all essential considerations that should be regarded. More research into field applications is required to evaluate the potential of chitosan nanoparticles on yield traits. 

## Figures and Tables

**Table 1:** The nucleotide sequences of primers utilized in this investigation.

Primer Name	Forward Primer (5′ . . . . . 3′)	Reverse Primer (5′ . . . . . 3′)
*PR1* (basic)	CTGGAGCACGAAGCTGCAG	CGAGTGCTGGAGCTTGCAGT
*PR2*	CTCGACATCGGTAACGACCAG	GCGGCGATGTACTTGATGTTC
*PR3*	AGAGATAAGCAAGGCCACGTC	GGTTGCTCACCAGGTCCTTC
*PR4*	CGAGGATCGTGGACCAGTG	GTCGACGAACTGGTAGTTGACG
*PR5*	ACAGCTACGCCAAGGACGAC	CGCGTCCTAATCTAAGGGCAG
*PR10*	TTAAACCAGCACGAGAAACATCAG	ATCCTCCCTCGATTATTCTCACG
*β-tubulin*	GCCATGTTCAGGAGGAAGG	CTCGGTGAACTCCATCTCGT

## Data Availability

Available upon request from the corresponding author.

## Supplementary Materials

The following supporting information can be downloaded at: www.mdpi.com/article/10.3390/jof8030304/s1, Supplementary Table S1: Wheat leaf rust infection types used in disease assessment for seedling stage according to Johnston and Browder.

## References

[1] Omara R.I., Shahin A.A., Ahmed S.M., Mostafa Y.S., Alamri S.A., Hashem M., Elsharkawy M.M. (2021). Wheat resistance to stripe and leaf rusts conferred by introgression of slow rusting resistance genes. J. Fungi.

[2] Nazim M., El-Shehidi A.A., Abdou Y.A., El-Daoudi Y.H. Yield loss caused by leaf rust on four wheat cultivars under epiphytotic levels. Proceedings of the 4th Conference of Microbiology.

[3] Ali R.G., Omara R.I., Ali Z.A. (2016). Effect of leaf rust infection on yield and technical properties in grains of some Egyptian wheat cultivars. Menoufia J. Plant Prot..

[4] Gad A.M., Abdel-Halim K.Y., Seddik F.A., Soliman H.M.A. (2020). Comparative of fungicidal efficacy against yellow rust disease in wheat plants in compatibility with some biochemical alterations. Menoufia J. Plant Prot..

[5] Omara R.I., Nehela Y., Mabrouk O.I., Elsharkawy M.M. (2021). The emergence of new aggressive leaf rust races with the potential to supplant the resistance of wheat cultivars. Biology.

[6] Xi K., Kumar K., Holtz M.D., Turkington T.K., Chapman B. (2015). Understanding the development and management of stripe rust in central Alberta. Can. J. Plant Pathol..

[7] Hafez Y.M., El-Baghdady N.A. (2013). Role of reactive oxygen species in suppression of barley powdery mildew fungus, *Blumeria graminis* f. sp. *hordei* with benzothiadiazole and riboflavin. Egypt J. Biol. Pest. Cont..

[8] Hafez Y.M., Soliman N.K., Saber M.M., Imbaby I.A., Abd-Elaziz A.S. (2014). Induced resistance against *Puccinia triticina* the causal agent of wheat leaf rust by chemical inducers. Egypt J. Biol. Pest. Cont..

[9] Omara R.I., Kamel S.M., Hafez Y.M., Morsy S.Z. (2015). Role of non-traditional control treatments in inducing resistance against wheat leaf rust caused by *Puccinia triticina*. Egypt J. Biol. Pest. Cont..

[10] Al-Juboory H., Al-Hadithy H. (2021). Evaluation of chitosan and salicylic acid to induce systemic resistance in eggplant against under plastic *Botrytis cinerea* house conditions. Indian J. Ecol..

[11] Omara R.I., Abdelaal K.A.A. (2018). Biochemical, histopathological and genetic analysis associated with leaf rust infection in wheat plants (*Triticum aestivum* L.). Physiol. Mol. Plant Pathol..

[12] El-Saadony M.T., Alkhatib F.M., Alzahrani S.O., Shafi M.E., El Abdel-Hamid S., Taha T.F., Aboelenin S.M., Soliman M.M., Ahmed N.H. (2021). Impact of mycogenic zinc nanoparticles on performance, behavior, immune response, and microbial load in Oreochromis niloticus. Saudi J. Biol. Sci..

[13] Bano A. (2020). Interactive effects of Ag-nanoparticles, salicylic acid, and plant growth promoting rhizobacteria on the physiology of wheat infected with yellow rust. J. Plant Pathol..

[14] Jasrotia P., Kashyap P.L., Bhardwaj A.K., Kumar S., Singh G.P. (2018). Scope and applications of nanotechnology for wheat production: A review of recent advances. Wheat Barley Res..

[15] El-Saadony M.T., Saad A.M., Najjar A.A., Alzahrani S.O., Alkhatib F.M., Shafi M.E., Selem E., Desoky E.-S., Fouda S.E.E., El-Tahan A.M. (2021). The use of biological selenium nanoparticles to suppress *Triticum aestivum* L. crown and root rot diseases induced by *Fusarium* species and improve yield under drought and heat stress. Saudi J. Biol. Sci..

[16] El-Saadony M.T., Sitohy M.Z., Ramadan M.F., Saad A.M. (2021). Green nanotechnology for preserving and enriching yogurt with biologically available iron (II). Innov. Food Sci. Emerg. Technol..

[17] Elizabath A., Babychan M., Mathew A.M., Syriac G.M. (2019). Application of nanotechnology in agriculture. Int. J. Pure Appl. Biosci..

[18] Elieh-Ali-Komi D., Hamblin M.R. (2016). Chitin and chitosan: Production and application of versatile biomedical nanomaterials. Int J Adv. Res..

[19] Kocięcka J., Liberacki D. (2021). The potential of using chitosan on cereal crops in the face of climate change. Plants.

[20] Abd El-Hack M.E., El-Saadony M.T., Shafi M.E., Zabermawi N.M., Arif M., Batiha G.E., Khafaga A.F., Abd El-Hakim Y.M., Al-Sagheer A.A. (2020). Antimicrobial and antioxidant properties of chitosan and its derivatives and their applications: A review. Int. J. Biol. Macromol..

[21] Omar H.S., Al Mutery A., Osman N.H., Reyad N.E.H.A., Abou-Zeid M.A. (2021). Genetic diversity, antifungal evaluation and molecular docking studies of Cu-chitosan nanoparticles as prospective stem rust inhibitor candidates among some Egyptian wheat genotypes. PLoS ONE.

[22] Pichyangkura R., Chadchawan S. (2015). Biostimulant activity of chitosan in horticulture. Sci. Hortic..

[23] Soleiman N.H., Solis I., Ammar A., Dreisigacker S., Soleiman M.H., Martinez F. (2014). Resistance to leaf rust in a set of durum wheat cultivars and landraces in Spain. J. Plant Pathol..

[24] Johnston C.O., Browder L.E. (1966). Seventh revision of the international register of physiologic races of *Puccinia recondita* f.sp. tritici. Plant Dis. Report.

[25] Nair K.R.S., Ellingboe A.H. (1962). A method of controlled inoculations with condiospores of *Erysiphe graminis* var. tritici. Phytopatholology.

[26] Reifshneider F.J.B., Bolitexa L.S., Occhiena E.M. (1985). Powdery mildew of melon (*Cucumis melo*) caused by *Sphaerotheca fuliginea* in Brazil. Plant Dis..

[27] Parlevliet J.E. (1975). Partial resistance of barley to leaf rust, *Puccinia hordei*. I. effect of cultivars and development stage on latent period. Euphytica.

[28] Parlevliet J.E., Kuiper H.J. (1977). Partial resistance of barley to leaf rust, *Puccinia hordei*. IV. Effect of cultivars and development stage on infection frequency. Euphytica.

[29] Menzies J.G., Belanger R.R. (1996). Recent advances in cultural management of diseases of greenhouse crops. Can. J. Plant Pathol..

[30] Aebi H. (1984). Catalase in vitro. Methods Enzymol..

[31] Hammerschmidt R., Nuckles E.M., Kuć J. (1982). Association of enhanced peroxidase activity with induced systemic resistance of cucumber to *Colletotrichum lagenarium*. Physiol. Plant Pathol..

[32] Huckelhoven R., Fodor J., Preis C., Kogel K.H. (1999). Hypersensitive cell death and papilla formation in barley attacked by the powdery mildew fungus are associated with hydrogen peroxide but not with salicylic acid accumulation. Plant Physiol..

[33] Nassar M.A., El-Sahhar K.F. (1998). Botanical Preparations and Microscopy (Microtechnique).

[34] Elsharkawy M., Derbalah A., Hamza A., El-Shaer A. (2020). Zinc oxide nanostructures as a control strategy of bacterial speck of tomato caused by *Pseudomonas syringae* in Egypt. Environ. Sci. Pollut Res..

[35] Desmond O.J., Edgar C.I., Manners J.M., Maclean D.J., Schenk P.M., Kazan K. (2006). Methyl jasmonate induced gene expression in wheat delays symptom development by the crown rot pathogen *Fusarium pseudograminearum*. Physiol. Mol. Plant Pathol..

[36] Livak K.J., Schmittgen T.D. (2001). Analysis of relative gene expression data using real-time quantitative PCR and the 2^−ΔΔCT^ method. Methods.

[37] Chen X.M. (2005). Epidemiology and control of stripe rust (*Puccinia striiformis* f sp. *tritici*) on wheat. Can. J. Plant Pathol..

[38] Hossard L., Philibert A., Bertrand M., Colnenne-David C., Debaeke P., Munier-Jolain N., Jeuffroy M.H., Richard G., Makowski D. (2015). Effects of halving pesticide use on wheat production. Sci. Rep..

[39] El Hadrami A., Adam L.R., El Hadrami I., Daayf F. (2010). Chitosan in plant protection. Mar. Drugs.

[40] Xu J., Zhao X., Han X., Du Y. (2007). Antifungal activity of oligo chitosan against *Phytophthora capsici* and other plant pathogenic fungi *in vitro*. Pestic. Biochem. Physiol..

[41] Ata A.A., El-Samman M.G., Moursy M.A., Mostafa M.H. (2008). Inducing resistance against rust disease of sugar beet by certain chemical compounds. Egypt J. phytopathol..

[42] Hassan O., Chang T. (2017). Chitosan for eco-friendly control of plant disease. Asian J. Plant Pathol..

[43] Benhamou N., Kloepper J.W., Tuzan B.B. (1998). Induction of resistance against Fusarium wilt of tomato by combination of chitosan with an endophytic bacterial stain: Ultrastructure and cytochemistry of host response. Planta.

[44] Ragab M.M., Ragab M.M., El-Nagar M.A., Farrag E.S. (2001). Effect of chitosan and its derivatives as an antifungal and preservative agent on storage of tomato fruits. Egypt J. Phytopathol..

[45] Bautista B.S., Hern N., Indez L., Bosquez M.E., Wil C.L. (2003). Effect of chitosan and plant extracts on growth *of Colletotrichum gloeosporioides*, anthracnose level and quality of papaya fruit. Crop Prot..

[46] Rabea E., Badawy M.T., Stevens C., Smagghe G., Steurbaut W. (2003). Chitosan as antimicrobial agent: Applications and Mode of action. Biomacromolecules.

[47] Verma S.K., Das A.K., Gantait S., Kumar V., Gurel E. (2019). Applications of carbon nanomaterials in the plant system: A perspective view on the pros and cons. Sci. Total Environ..

[48] Catherine B., Marc O., Philippe T., Jacques D. (2004). Cloning and expression analysis of cDNAs corresponding to genes activated in cucumber showing systemic acquired resistance after BTH treatment. BMC Plant Biol..

[49] Yang S., Gaojian L. (2020). ROS and diseases: Role in metabolism and energy supply. Mol. Cell. Biochem..

[50] Xing K., Zhu X., Peng X., Qin S. (2015). Chitosan antimicrobial and eliciting properties for pest control in agriculture: A review. Agron. Sustain. Dev..

[51] Christian N., Jean P.M. (1999). Salicylic acid induction defiant mutants of *Arabidiopsis* express *PR*-2 and *PR-5* and accumulate high levels of camalexin after pathogen inoculation. Plant Cell.

[52] Omara R.I., Essa T.A., Khalil A.A., Elsharkawy M.M. (2020). A case study of non-traditional treatments for the control of wheat stem rust disease. Egypt J. Biol. Pest. Cont..

[53] Quiroga M., Guerrero C., Botella M.A., Barcelo A., Amaya I., Meding M., Alonso F.J., Forcheti S.M., Tigir H., Vulpuesta V.A. (2000). Tomato peroxidase involved in the synthesis of lignin and suberin. Plant Physiol..

[54] Bruno M.M., Ulrik N., Lilliane G., Hans-joachim R. (1990). Specific inhibition of lignifications breaks hypersensitive resistance of wheat to stem rust. Plant Physiol..

[55] Cohen Y. (2001). The BABA story of induced resistance. Phytoparasitica.

[56] Chang M.M., Hadwiger L.A., Horovits D. (1992). Molecular characterization of a pea 1,3 glucanase induced by *Fusarium solani* and chitosan challenge. Plant Mol. Biol..

[57] Siddaiah C.N., Prasanth K.V.H., Satyanarayana N.R., Mudili V., Gupta V.K., Kalagatur N.K., Satyavati T., Dai X.-F., Chen J.-Y., Mocan A. (2018). Chitosan nanoparticles having higher degree of acetylation induce resistance against pearl millet downy mildew through nitric oxide generation. Sci. Rep..

[58] Liu R., Wang Z.-Y., Li T.-T., Wang F., An J. (2014). The role of chitosan in polyphenols accumulation and induction of defense enzymes in *Pinus koraiensis* seedlings. Chin. J. Plant Ecol..

