# Supplementary material for: Mechanism of Wheat Leaf Rust Control Using Chitosan Nanoparticles and Salicylic Acid
Source: J Fungi (Basel). 2022 Mar 16;8(3):304. doi: 10.3390/jof8030304 (PMC8950986; doi:10.3390/jof8030304)
Supplement: Supplementary file 1 [file jof-08-00304-s001.zip › jof-1615638-supplementary.pdf]

Supplementary Table S1. Wheat leaf rust infection types used in disease assessment for seedling stage according to Johnston and Browder [20].

Host response (class)	Infection type	Disease symptoms
**Immune**	0	No uredia or other macroscopic sign of infection.
**Nearly immune**	0;	No uredia but hypersensitive necrotic or chlorotic flecks present.
**Very resistant**	1	Small uredia surrounded by necrosis.
**Moderately resistant**	2	Small to medium uredia surrounded by chlorosis or necrosis.
**Moderately susceptible**	3	Medium-sized uredia that may be associated with chlorosis.
**Very susceptible**	4	Large uredia without chlorosis or necrosis or rarely necrosis.
**Heterogeneous**	X	Random distribution of different variable sized uredia on single leaf.
